# Optical Imaging of PARP1 in Response to Radiation in Oral Squamous Cell Carcinoma

**DOI:** 10.1371/journal.pone.0147752

**Published:** 2016-01-25

**Authors:** Susanne Kossatz, Wolfgang A. Weber, Thomas Reiner

**Affiliations:** 1 Department of Radiology, Memorial Sloan Kettering Cancer Center, New York, New York, 10065, United States of America; 2 Department of Radiology, Weill Cornell Medical College, New York, New York, 10065, United States of America; University of South Alabama Mitchell Cancer Institute, UNITED STATES

## Abstract

Targeting and inhibiting DNA repair pathways is a powerful strategy of controlling malignant growth. One such strategy includes the inhibition of PARP1, a central element in the intracellular DNA damage response. To determine and visualize the expression and intercellular distribution of PARP1 *in vivo*, and to monitor the pharmacokinetics of PARP1 targeted therapeutics, fluorescent small probes were developed. To date, however, it is unclear how these probes behave in a more realistic clinical setting, where DNA damage has been induced through one or more prior lines of therapy. Here, we use one such imaging agent, PARPi-FL, in tissues both with and without prior DNA damage, and investigate its value as a probe for PARP1 imaging. We show that PARP1 expression in oral cancer is high, and that the uptake of PARPi-FL is selective, irrespective of whether cells were exposed to irradiation or not. We also show that PARPi-FL uptake increases in response to DNA damage, and that this increase is reflected in higher enzyme expression. Our findings provide a framework for measuring exposure of cells to external beam radiation, and could help to elucidate the effects of such treatments non-invasively in mouse models of cancer.

## Introduction

Oral cancer is a type of malignant growth that more than 45,000 individuals will be diagnosed with in the United States in 2015 alone. Treatment options have improved over the last years, and the overall 5-year relative survival rates have increased from 52.7% in 1975 to 66.3% in 2007. This is in part due to the introduction of novel treatment options, one of which is intensity-modulated radiation therapy (IMRT) [1–4]. This type of radiation therapy allows the administration of ionizing radiation with varying intensities, effectively depositing DNA-damage events in a fairly defined region [5] of the oral cavity. While IMRT is administered routinely, little is known about the spatial resolution of DNA damage on a case-by-case basis, and whether this damage can be visualized using injectable probes. Such an agent could ultimately aid preclinical investigations and improve the precision and methodology of IMRT in the clinic.

With this in mind, we turned our focus to PARP1, an enzyme that is instrumental for DNA damage response after single and double strand DNA breaks [6–8]. PARP1 stands at the beginning of the DNA repair cascade, and its activation leads to the recruitment of other DNA damage response factors [9–15]. The activation of PARP1 after DNA damage is quick, and long branched poly(ADP-ribose) chains are formed seconds after the event [16–18]. In this study, we sought to measure the expression of PARP1 using the imaging agent PARPi-FL. The fluorescent small molecule inhibits PARP1 action by binding to the enzyme’s ADP binding pocket, similar to therapeutic small molecules [19, 20].

PARP1 is a desirable imaging target, because it is overexpressed in a wide variety of cancers [21–29], and its overexpression allows the differentiation of tumor cells from healthy surrounding and invaded tissues [30]. PARPi-FL has been validated [31–33] as an imaging agent for tumor tissue, but its use for tissues that underwent treatment has not been investigated. The questions we aimed to address in this study are the following: (A) does PARPi-FL accumulate selectively in tumor nuclei, even after delivering a dose of radiation lethal to >95% of a tumor cell population? (B) Is the marker distributed and retained in tumor tissue, even after delivery of a therapeutic dose of radiation? (C) Do PARP1 levels respond to ionizing radiation, and can this response be imaged using PARPi-FL?

The understanding of where, how and to what extent radiation damage unfolds is critical to designing effective and optimized treatments regimens. The correlation between irradiation and DNA damage in oral cancer cells has been shown on the histological level, for example by measuring phosphorylated γH2AX foci formation [34, 35], but an injectable marker which can image such a response is to date an unmet clinical need. Here, we show that PARP1 is overexpressed in oral cancer, and using this model, we determine that PARPi-FL is a selective marker in oral cancer cell lines, irrespective of whether they received ionizing radiation or not. Our results show that PARPi-FL uptake increases as a response to ionizing radiation within the first 48 hours. We also show that the elevated uptake correlates with higher PARP1 expression, and that uptake is selective not only *in vitro*, but also *in vivo*. The studies described here provide a framework for further investigating PARP1 as a marker of radiation-induced DNA damage.

## Methods

### Cell Culture

Experiments were carried out using two human OSCC cell lines. FaDu (hypopharyngeal SCC; ATCC, Manassas, VA) were maintained in MEM medium and Cal27 cells (tongue SCC; ATCC, Manassas, VA) were maintained in D-MEM medium, both containing 10% (v/v) FBS and 1% Penicillin/Streptavidin. Cells were grown in monolayer culture at 37°C in a 5% CO_2_ humidified atmosphere and passaged at 70–80% confluency.

### Animal models

Female athymic nude (NCr-Foxn1nu, Taconic, Hudson, NY) were housed under standard conditions with water and food ad libitum. Animals were anesthetized with 2% isoflurane throughout tumor implantation, irradiation and imaging. To implement subcutaneous human OSCC tumors, 2×10^6^ FaDu or Cal27 cells were dispensed in 50 μl medium, and 50 μl Matrigel^™^ (BD Biosciences, Bedford, MA) was added before injection on the lower back of the animals. For irradiation experiments, we used bilateral FaDu xenografts. Experiments were started 15 days after xenografting, when tumors had reached 100–150 mm^3^ volume. All animal experiments were done in accordance with institutional guidelines and approved by the IACUC of MSK and followed NIH guidelines for animal welfare. Animals were sacrificed before the experimental endpoint if tumors reached a volume of more than 1000 mm^3^, or animals displayed severe signs of distress such as rapid weight loss, crouching and impaired movement.

### PARP1 expression in tissues

PARP1 antigen expression was assessed in mouse tongue, FaDu and Cal27 xenografts using IHC to determine their basic PARP1 expression before irradiation. The staining was done using the Discovery XT processor (Ventana Medical Systems, Tucson, AZ). Paraffin-embedded formalin fixed 3 μm sections were deparaffinized with EZPrep buffer, antigen retrieval was performed with CC1 buffer (both Ventana Medical Systems, Tucson, AZ) and sections were blocked for 30 min with Background Buster solution (Innovex, Richmond, CA). The anti-PARP1 rabbit polyclonal antibody (sc-7150, Santa Cruz Biotechnology, Santa Cruz, CA) was incubated for 5 h (0.2 μg/ml), followed by 1 hour incubation with biotinylated goat anti-rabbit IgG (PK6106, Vector Labs, Burlingame, CA) at a 1:200 dilution. For detection, a DAB detection kit (Ventana Medical Systems, Tucson, AZ) was used according the manufacturer instructions. Sections were counterstained with hematoxylin and coverslipped with Permount (Fisher Scientific, Pittsburgh, PA, USA). Incubating with a rabbit IgG instead of the primary antibody controlled for non-specific binding of the secondary antibody. Adjacent sections were stained with hematoxylin and eosin for morphological evaluation of the tissue. The staining was performed at the Molecular Cytology Core Facility of MSK. For quantification of PARP1 protein distribution, thresholding was performed (MetaMorph^®^ Software, Molecular Devices, Sunnyvale, CA) on brown (PARP1) and blue (tissue) areas of digitalized sections and the relative PARP1 positive area was calculated by dividing the brown area by the total tissue area. 10 field-of-views were analyzed per section.

### Cell irradiation and clonogenic survival

Cells were irradiated with 0, 2, 4, 6, 8 and 10 Gy in 75 cm^2^ culture flasks using a J.L. Shepherd Cesium irradiator (J.L. Shepherd, San Fernando, CA) at a dose rate of 174 cGy/min. Clonogenic survival was assessed as previously described [36]. Briefly, after irradiation, cells were trypsinized, counted and pre-defined numbers of viable cells were plated in 6-Well plates in triplicate. In order to receive a sufficient colony count (between 50 and 100), two cell numbers were plated per irradiation dose (0 Gy: 200, 500; 2 Gy: 500, 1000; 6 Gy: 800, 3000; 8 Gy: 1600, 7000; 10Gy: 2500, 8000). Cells were cultured 10–14 days and then stained with 0.5% Crystal Violet (Sigma-Aldrich, St. Louis, MO) for 10 min at room temperature. Only colonies consisting of at least 50 cells were counted and a mean was calculated from the triplicate wells. The plating efficiency of each irradiation dose was calculated by dividing the number of counted colonies by the number of cells plated. The relative clonogenic survival was calculated by dividing the plating efficiency of a certain irradiation dose by the plating efficiency of untreated cells. Three independent experiments were carried out for each cell line.

### PARPi-FL uptake of cells

To determine the binding of PARPi-FL to cells, they were plated in 8-Well Chamber Slides (Lab-Tek Brand; Nalge Nunc International, Naperville, IL). After 24 hours, cells were treated with 0 or 10 Gy irradiation in a J.L. Shepherd Cesium irradiator (J.L. Shepherd, San Fernando, CA) at a dose rate of 174 cGy/min. 24 hours post irradiation, cells were incubated with a 1 μM solution of PARPi-FL for 20 min at 37°C, followed by two 5 min incubations in full medium and one wash in PBS. Subsequently, cells were fixed with 4% Paraformaldehyde, plastic chambers were removed and slides were mounted with Mowiol^®^ mounting medium containing Hoechst 33342 for counterstaining of cell nuclei. Imaging was done using a Leica SP5 upright confocal microscope (Leica, Buffalo Grove, IL), equipped with appropriate lasers and emission filters. PARPi-FL was imaged using the FITC channel and 488 nm laser excitation.

### Effect of cell irradiation on PARPi-FL uptake

We quantified the change in PARPi-FL uptake in FaDu and Cal27 cells after irradiation using Flow Cytometry. First, cells were irradiated with 0, 2, 4 and 10 Gy in 25 cm^2^ culture flasks using the J.L. Shepherd Cesium irradiator (J.L. Shepherd, San Fernando, CA) at a dose rate of 174 cGy/min. At different time intervals post irradiation (6, 24 and 48 hours) PARPi-FL staining was initiated. Following a wash with PBS, cells were trypsinized, counted and portions of 0.5 × 10^6^ cells of the single cell suspension were aliquoted into 1.5 ml Eppendorf tubes (Eppendorf, Hamburg, Germany). For each time point and irradiation dose, samples were either left unstained, were stained with PARPi-FL or Olaparib/PARPi-FL. Co-incubation with a 10-fold excess of the non-fluorescent PARP1 inhibitor Olaparib was carried out to control for binding specificity of PARPi-FL. For staining, cells were washed with 1 ml FACS buffer (1% BSA (w/v) in PBS). Then, 1 ml of the staining solution (FACS buffer only, 0.5 μM PARPi-FL in FACS buffer or 5 μM Olaparib/0.5 μM PARPi-FL in FACS buffer) was added for 20 min at 37°C, followed by one 5 min wash in 1 ml FACS buffer. Next, cells were centrifuged, the supernatant was aspirated and cells were resuspended in 0.5 ml FACS buffer and transferred to 5 ml round bottom flow cytometry tubes (BD Biosciences, Bedford, MA) through a 40 μm strainer to remove doublets and left on ice until measurement in the flow cytometer (LSR^®^ II, BD Biosciences, Bedford, MA). For each measurement, 10,000 events were counted. Raw data were processed in FlowJo software in order to calculate the changes in PARPi-FL uptake after irradiation. Cell clumps and debris were eliminated using the corresponding gates (forward and side scatter) for the unstained cell population. The gates were applied to all stained samples. PARPi-FL fluorescence was imaged in the FITC channel against side scatter (area).

### *In vivo* irradiation

Bilateral FaDu xenografts were inoculated 15 days before irradiation on the left and right side of the lower back of female athymic nude mice (n ≥ 3/group). Tumor volume was measured with a caliper every 3–5 days and calculated by the formula π/6 × (length × width × height of the tumor). Tumors on the right side were irradiated with 10 Gy using an image-guided microirradiator (X-Rad 225 Cx, Presicion X-Ray, North Branford, CT). The irradiation area was centered on the tumor by using the built-in cone-beam CT for soft tissue imaging and a 2×2 cm collimator. X-Ray irradiation was delivered at a dose rate of 3.1306 Gy/min while animals were under 2% isoflurane anaesthesia.

### PARP1 expression following irradiation

At 24 hours and 48 hours after the irradiation, animals were sacrificed using carbon dioxide asphyxiation. Tumors were explanted, formalin-fixed and embedded in paraffin for immunofluorescent PARP1 staining. This was done following the protocol for IHC as described above, with the difference that detection was performed with Streptavidin-HRP D (from DABMap Kit, Ventana Medical Systems), followed by incubation with Tyramide Alexa Fluor 594 (T20935, Invitrogen, Carlsbad, CA), prepared according to the manufacturer’s instructions. Sections were counterstained with 4’,6-diamidino-2-phenylindole (DAPI) for 10 min and coverslipped with Mowiol^®^ mounting medium (Sigma-Aldrich, St. Louis, MO). Immunofluorescence staining allowed for evaluation of the intensity of the PARP1 signal in each nucleus in addition to the PARP1 positive area. In each section, 10 fields of view were analyzed (total area 3.64 mm^2^). For each tumor, three sections were analyzed. Per group (irradiated and non irradiated) 4 tumors were analyzed.

The PARP1 quantification was done on digitalized slides using an automated segmentation and quantification protocol generated with the software MetaMorph^®^ (Molecular Devices, Sunnyvale, CA) using the three scanned channels (red = PARP1, blue = DAPI, green = autofluorescence). The PARP1 positive area was determined by thresholding the red fluorescent area and dividing it by the whole tissue area, which was determined based on autofluorescence. PARP1 intensity was determined by measuring the red fluorescence intensity in all nuclei, which were thresholded using DAPI staining. The measured fluorescence intensities were averaged over all nuclei in each field-of-view.

### Synthesis of PARPi-FL

Synthesis of the optical imaging agent PARPi-FL was carried out analogous to what was previously described [32]. Briefly, the green fluorescent dye BODIPY-FL NHS-ester (Invitrogen, Carlsbad, CA) was conjugated to 4-(4-fluoro-3-(piperazine-1-carbonyl)benzyl)phthalazin-1(2H)-one, followed by purification via preparative HPLC (Waters’ XTerra C-18 5 μm column, 7 ml/min, 5% to 95% of acetonitrile in 15 min). PARPi-FL was obtained in 70–79% yield as a red solid in >97% purity. The identity of PARPi-FL was confirmed using ESI-MS (MS(+) m/z = 663.4 [M+Na]+). For *in vivo* imaging studies, PBS (117 μl) was slowly added to an aliquot of PARPi-FL (50 μg, 75 nmol) in 50 μl of poly(ethylene glycol) (PEG300, Sigma-Aldrich, St. Louis, MO) to obtain a final injection volume of 167 μl.

### Imaging of PARPi-FL uptake in response to irradiation

Cohorts of subcutaneous FaDu tumor bearing animals were injected intravenously with PARPi-FL (75 nmol/167 μl PBS with 30% PEG300) 24 hours and 48 hours post irradiation and 90 min before sacrifice by carbon dioxide asphyxiation. Irradiated and non-irradiated tumors as well as tongues were explanted and the fresh tissues imaged immediately in the epifluorescence system IVIS (PerkinElmer, Waltham, MA) using the standard filter set for GFP imaging. Autofluorescence was removed using spectral unmixing. The PARPi-FL signal was analyzed semiquantitatively by measuring the average radiant efficiency [p/s/cm2/sr]/[μW/cm^2^] in regions of interest (ROIs) that were placed on the tissue under white light guidance. Resulting numbers are normalized for the integration time, binning, f/stop, field of view, illumination intensity, and the ROI area, making measurements comparable among each other. After epifluorescence imaging, the freshly excised whole tumors were imaged microscopically. Tissues were placed on a cover slip with a freshly cut surface facing the cover slip and images were taken on an inverted laser scanning confocal microscope using 488 nm laser excitation (LSM 5-Live, Zeiss, Jena, Germany). PARPi-FL stained tumors were also compared to tumors that did not receive PARPi-FL injection to assess the extent of autofluorescence in the images. To confirm the specificity of the PARPi-FL stain to PARP1 protein, cryosections of the excised tumors were stained with an anti-PARP1 antibody. For this, 10 μm cryosections were fixed with 4% Paraformaldehyde, blocked for 30 min with 3% goat serum, stained overnight with the primary antibody (rabbit anti-PARP1, 1μg/ml, sc-7150, Santa Cruz), rabbit IgG (isotype control) or antibody dilution buffer (no primary control, PBS containing 1%(w/v) BSA and 0.3% TritonX-100). This was followed by secondary antibody staining (goat anti-rabbit-AF680, 2μg/ml, Invitrogen), Slides were mounted with Mowiol (Sigma-Aldrich) containing Hoechst 33342 for nuclear counterstaining.

### Statistical analysis

Statistical analysis was performed using GraphPad Prism 6. Unless otherwise stated, data points represent mean values, and error bars represent standard deviations of biological replicates. P values were calculated using a Student’s unpaired t-test, corrected for multiple comparisons by the Holm-Sidak method with an alpha of 0.05 or 0.01 as the cutoff for significance.

## Results

### PARP1 expression in tissues

**Fig 1:**
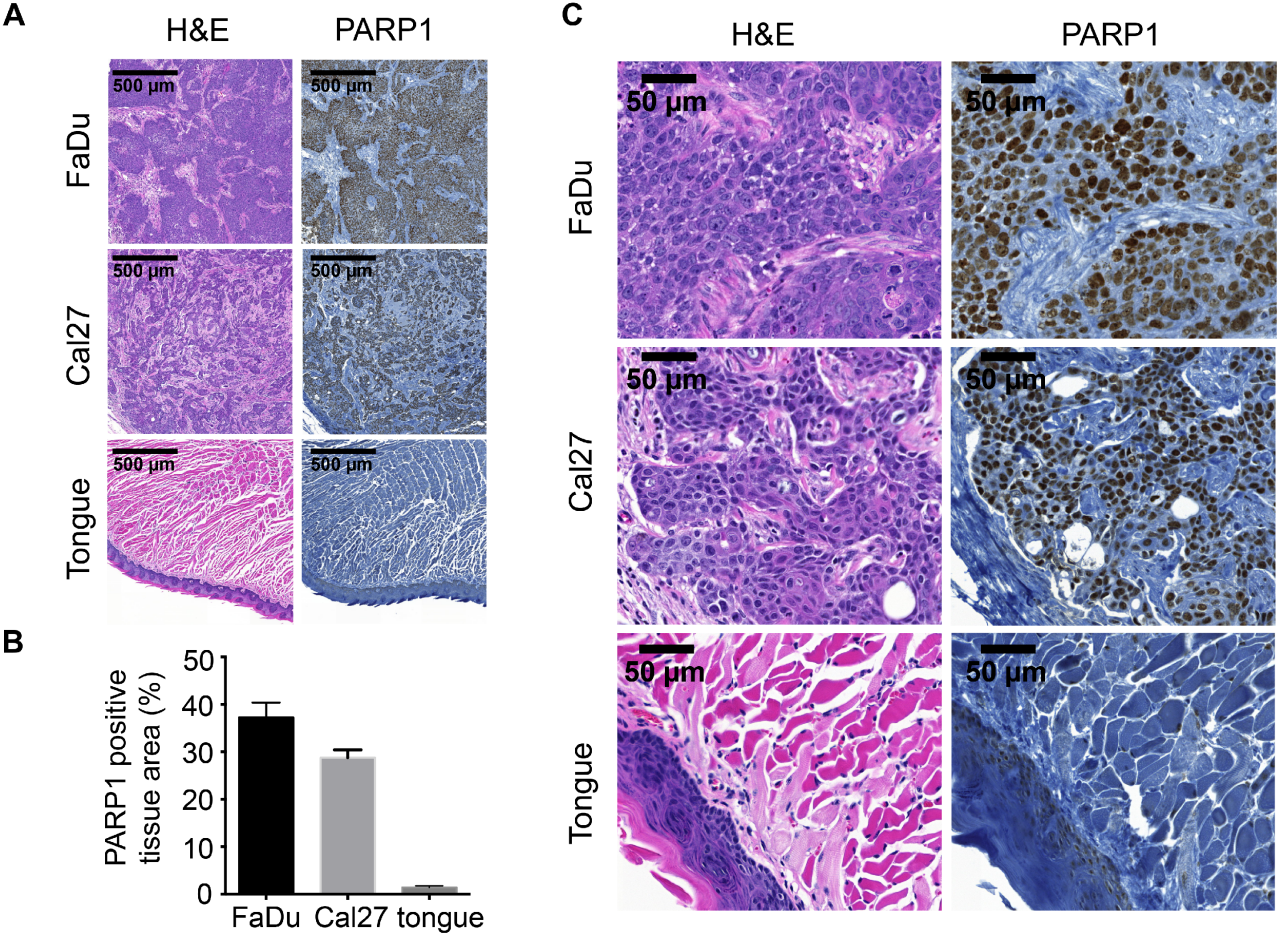
PARP1 expression in oral squamous cell carcinoma. (**A**) PARP1 Immunohistochemical staining of FaDu and Cal27 xenografts as well as normal mouse tongue and H&E staining of adjacent sections. (**B**) Quantification of the PARP1 positive area in FaDu, Cal27 and tongue tissue. (**C**) High magnification images of specimens displayed in (**A**).

We observed a strong nuclear expression of PARP1 in FaDu and Cal27 tumor tissue, but not in mouse tongue tissue (Fig 1A). This was quantified by measuring the percentage of tissue area that was stained by PARP1 (brown staining) compared to the whole tissue area (stained with hematoxylin; blue) using color thresholding. In FaDu tumors, 37.2 ± 3.2% of the tissue was found to be positive for PARP1, and Cal27 tumors displayed PARP1 staining in 28.7 ± 1.7% of the tissue. In contrast, tongue tissue (muscle and mucosa) had a 1.4 ± 0.4% PARP1 positive area (Fig 1B). Specificity of the staining became obvious in higher magnifications, where it could be seen that only tumor cell nuclei displayed strong PARP1 staining, but not stromal tissue or muscle tissue (Fig 1C).

### Cell survival and PARPi-FL imaging after external beam irradiation

**Fig 2:**
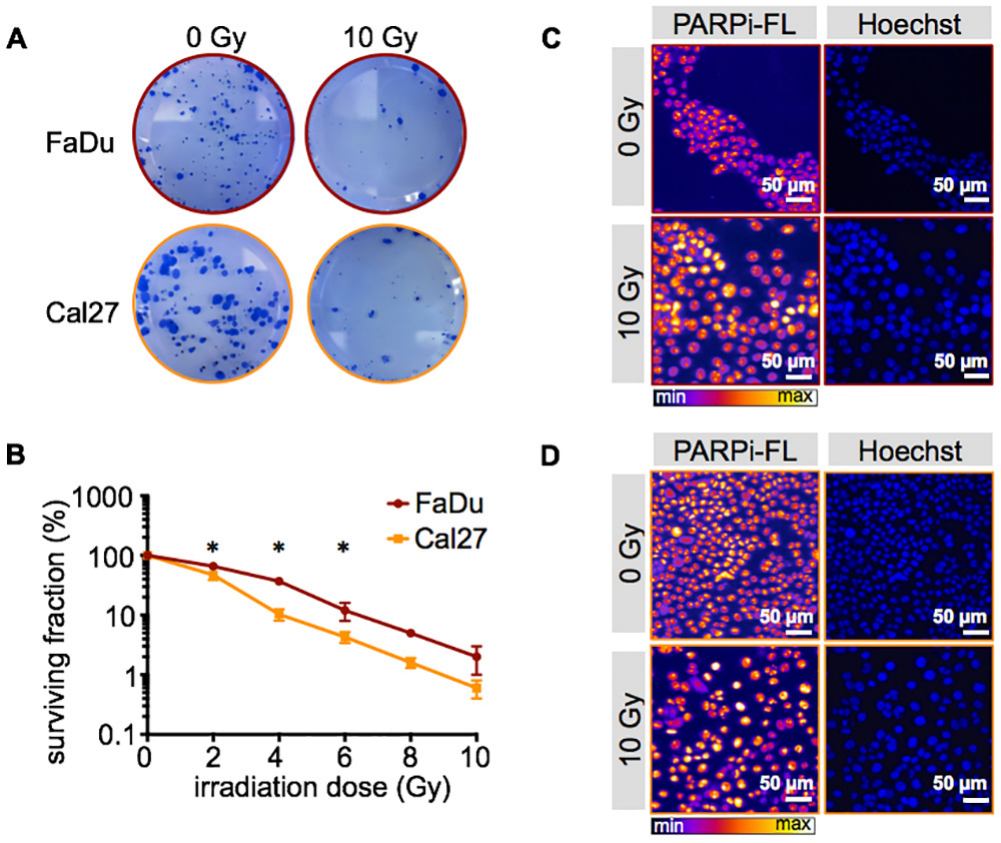
Effects of irradiation on cell survival and PARPi-FL uptake. (**A**) Examples of clonogenic growth of FaDu and Cal27 cells without (0 Gy) and after 10 Gy irradiation. (**B**) Clonogenic survival curve of FaDu and Cal27 cells after 0, 2, 4, 6, 8 and 10 Gy irradiation. Means ± SEM of three independent experiments with three parallels each. The asterisk indicates a p-value < 0.05 between FaDu and Cal27 cells using Student’s t-test. (**C**) Nuclear PARPi-FL uptake without and after 10 Gy irradiation, as observed microscopically in FaDu cells and (**D**) Cal27 cells. Nuclear localization was confirmed by Hoechst DNA stain. Representative images from n = 3 experiments.

Before imaging the PARPi-FL uptake in response to external beam irradiation in HNSCC cell lines, we quantified the effect of irradiation on cell survival. Clonogenic assays revealed that it decreased exponentially with increasing irradiation doses, as seen by reduction of the colony count (Fig 2B and 2C). At 2, 4 and 6 Gy, Cal27 cells were more sensitive to irradiation than FaDu cells, reflected by a significantly lower surviving fraction (p < 0.05, Fig 2B). The most pronounced difference could be seen at 4 Gy, where 37 ± 5% of FaDu cells, but only 10 ± 2% of Cal27 cells maintained their clonogenicity. At 10 Gy, the surviving fraction was 2 ± 1% for FaDu cells and 0.6 ± 0.2% for Cal27 cells, confirming the near-quantitative lethality of the external beam irradiation at this dose.

### Effect of cell irradiation on PARPi-FL uptake

PARP1 expression of FaDu and Cal27 cells was imaged using the fluorescent PARP1 inhibitor PARPi-FL. A quantitative relation between PARP1 expression and PARPi-FL binding has been shown previously [31, 32]. PARPi-FL accumulated in the nuclei of FaDu and Cal27 cells, irrespective of the fact whether cells were irradiated with 10 Gy or not (Fig 2C and 2D).

**Fig 3:**
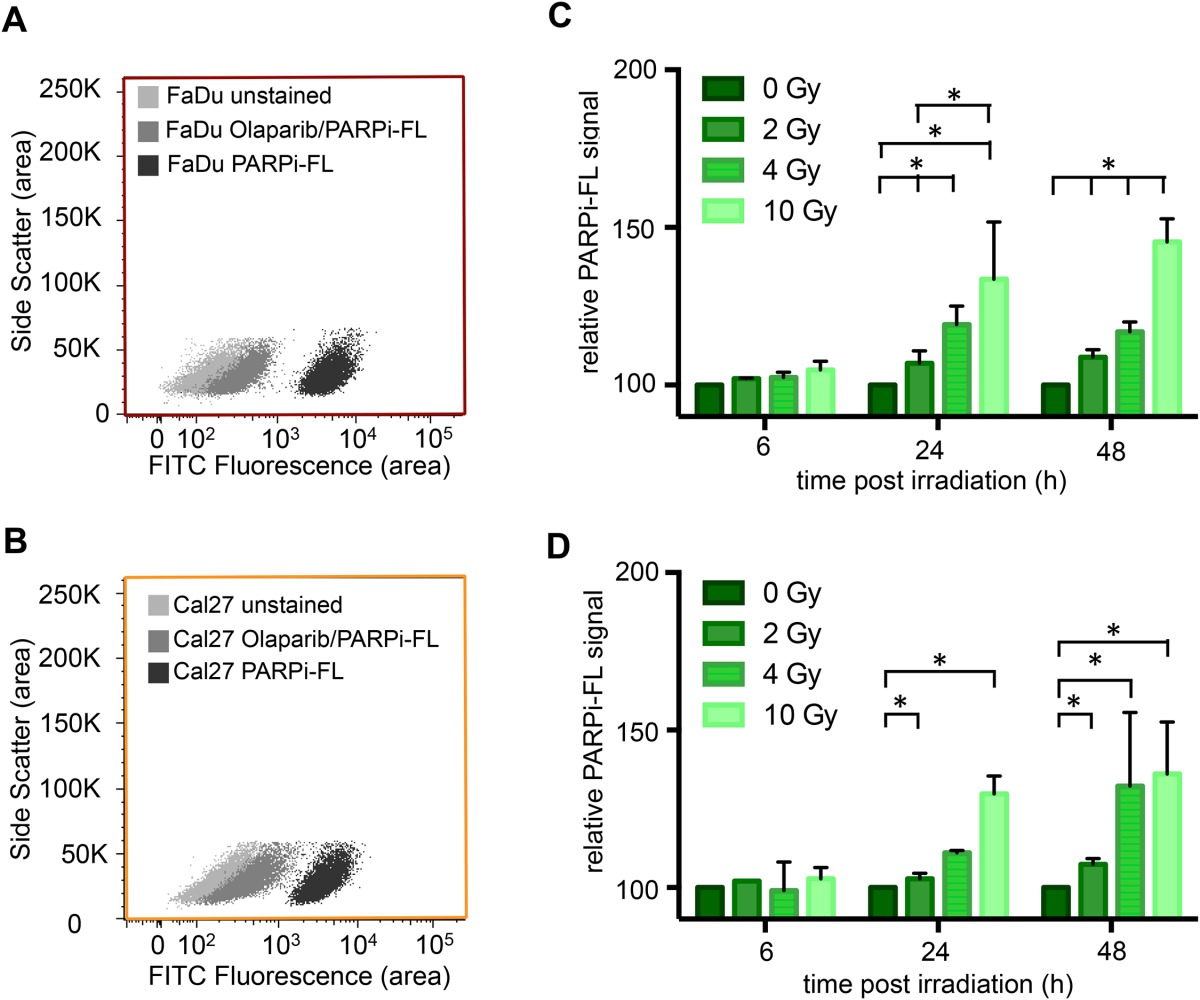
Quantification of PARPi-FL uptake after irradiation. Specific uptake of PARPi-FL into FaDu (**A**) and Cal27 (**B**) cells was determined by flow cytometry after incubation of cells with PARPi-FL or Olaparib/PARPi-FL. Olaparib competes with PARPi-FL for specific binding sites on PARP1. PARPi-FL uptake of FaDu (**C**) and Cal27 cells (**D**) after irradiation with 2, 4 and 10 Gy was determined and compared to 0 Gy. Mean PARPi-FL fluorescence signal was measured via flow cytometry in the FITC channel. Bars represent means ± SEM from three independent experiments with three parallels each.

To quantify the impact of PARP1 expression and PARPi-FL uptake, *in vitro* assays were performed at different time points and a set of irradiation doses. In non-irradiated cells, both cell lines showed a strong uptake of PARPi-FL (measured in the FITC channel), which separated the PARPi-FL incubated population from an unstained cell population (Fig 3A and 3B). We were able to almost completely suppress the imaging agent uptake, if the non-fluorescent PARP1 inhibitor Olaparib was co-incubated with PARPi-FL (50-fold excess). This shows specificity of PARPi-FL, because Olaparib and PARPi-FL, which are both derived from the same scaffold, compete for the ADP binding site on PARP1. Cells were irradiated with 2, 4 and 10 Gy and PARPi-FL uptake was quantified 6, 24 and 48 hours after the irradiation. At each time point, the mean fluorescence intensity was compared to non-irradiated cells (0 Gy) and the relative PARPi-FL signal was calculated.

In FaDu cells, no statistically significant differences between cell populations receiving different radiation doses were observed 6 hours post treatment. At 24 and 48 hours, however, all irradiation doses led to an increase in PARPi-FL uptake compared to the non-irradiated cells (Fig 3C). The strongest increase of PARPi-FL uptake was observed 48 hours after 10 Gy irradiation, where the PARPi-FL signal was 145.4 + 7.3% compared to non-irradiated cells.

Comparable results were observed in Cal27 cells. At 6 hours post irradiation, the PARPi-FL uptake was comparable for all cell populations. At 24 and 48 hours post irradiation, however, the PARPi-FL signal gradually increased with increasing irradiation doses (Fig 3D). The most pronounced effect was again observed at 10 Gy irradiation and 48 hours post treatment (136.0 + 16.6% compared to non-irradiation cells). When compared to FaDu cells, 4 Gy irradiation caused a stronger increase in PARPi-FL uptake 48 hours post irradiation.

### Effect of *in vivo* irradiation on PARP1 expression and PARPi-FL uptake

**Fig 4:**
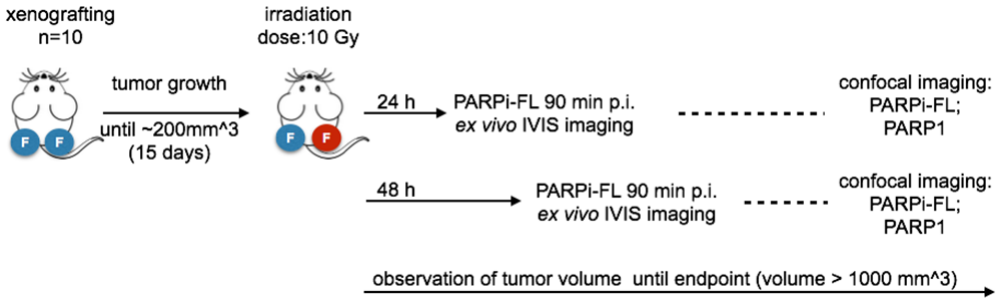
Experimental design for *in vivo* irradiation. Bilateral subcutaneous FaDu xenografts were grown for 15 days before 10 Gy irradiation of the tumor on the right flank. Subsequently, the effect of the irradiation on PARP1 expression and PARPi-FL uptake was observed 24 hours and 48 hours post irradiation and compared between the non-irradiated and the irradiated tumor. The effect of irradiation on tumor growth was monitored until day 26 post tumor inoculation or until tumors exceeded a volume of 1000 mm^3^.

**Fig 5:**
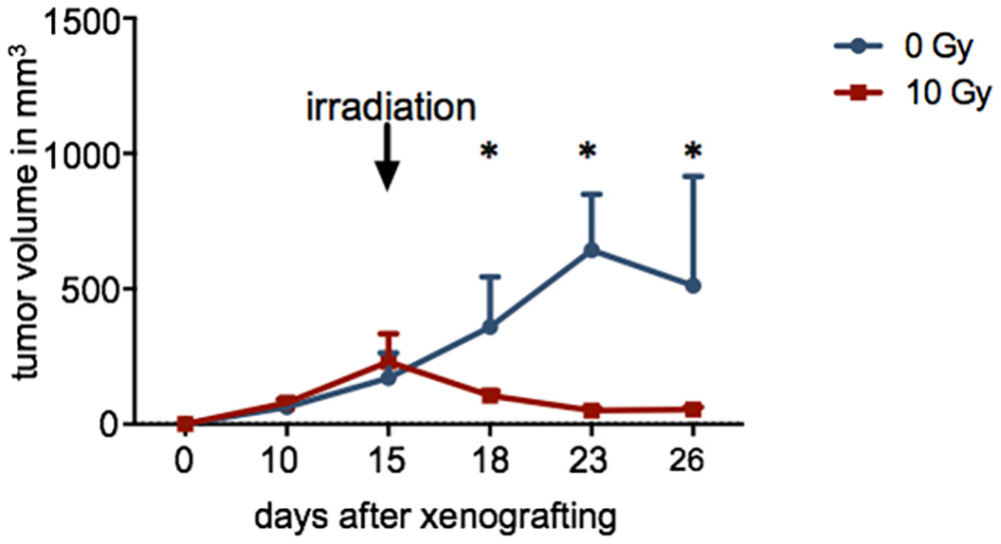
Growth curves of subcutaneous FaDu tumors over 26 days. Tumors were irradiated with 10 Gy using an image-guided microirradiator (day 15). Controls (0 Gy) were not irradiated (n≥4/group). The asterisk indicates statistical significance with p < 0.05 using the Student’s t-test.

The effect of 10 Gy irradiation on PARP1 expression and PARPi-FL uptake was assessed in bilateral FaDu tumor bearing nude mice, where the tumor on the right flank was exposed to 10 Gy using an image-guided microirradiator on day 15 after tumor inoculation (Fig 4). The impact of irradiation was also monitored by looking at the tumor volume for up to 26 days after the tumor inoculation (Fig 5). In the non-irradiated tumors, the volume gradually increased from 171 ± 92 mm^3^ on day 15 to 512 ± 403 mm^3^ until the end of the observation. Irradiation, on the other hand, led to an immediate reversal of the tumor growth curve and a decrease of mean tumor volume from 230 ± 327 mm^3^ on day 15 to 54 ± 14 mm^3^ on day 26.

**Fig 6:**
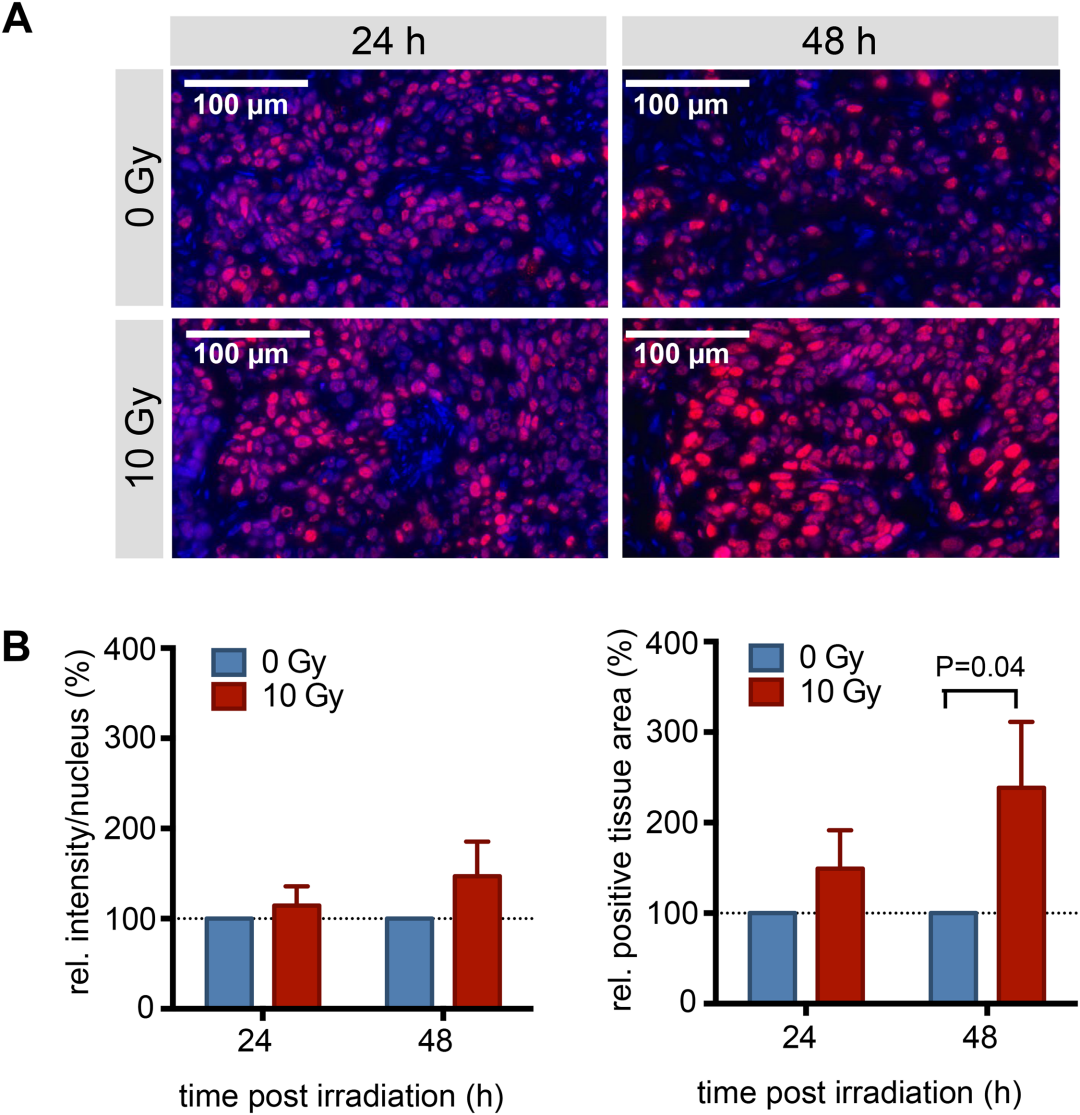
PARP1 Immunofluorescence staining of irradiated and non-irradiated tumors. (**A**) Representative PARP1 Immunofluorescence staining (n = 4/group). Red: PARP1; blue: Hoechst DNA stain. (**B**) Quantification of the PARP1 intensity per nucleus and the PARP1 positive area of irradiated (10 Gy) tumors in relation to non-irradiated (0 Gy) tumors. Values are based on quantification in 10 fields-of-view per tumor and four tumors per time point. Displayed are means with SEM (normalization was done for each individual before calculating the means).

Tumor sections of irradiated and non-irradiated tumors were stained for PARP1 using Immunofluorescence staining at 24 and 48 hours post irradiation (Fig 6A). Analogous to the *in vitro* experiments, an increased expression of PARP1 was observed in tissues that had been exposed to 10 Gy leading to higher PARP1 expression levels in individual nuclei (Fig 6B), with relative intensities of 114 ± 21% (24 hours post irradiation) and 147 ± 38% (48 hours post irradiation) compared to non-irradiated (0 Gy) tumor tissues. The PARP1 positive area was increased to a much higher degree than PARP1 expression, an effect that might be amplified by the influx of immune cells, which can themselves produce high levels of PARP1. After 24 hours, the PARP1 positive area was increased to 150 ± 42%, compared to non-irradiated tumors. After 48 hours, the PARP1 positive area more than doubled (239 ± 73%, p = 0.04) in irradiated vs. non-irradiated tumors (Fig 6B). Irradiation led to an increase in both PARP1 expression and density in FaDu tumors. We confirmed specificity of the staining at all observed time points and irradiation doses by using a rabbit IgG as primary antibody, which did not lead to nuclear or non-nuclear staining (S1 Fig).

**Fig 7:**
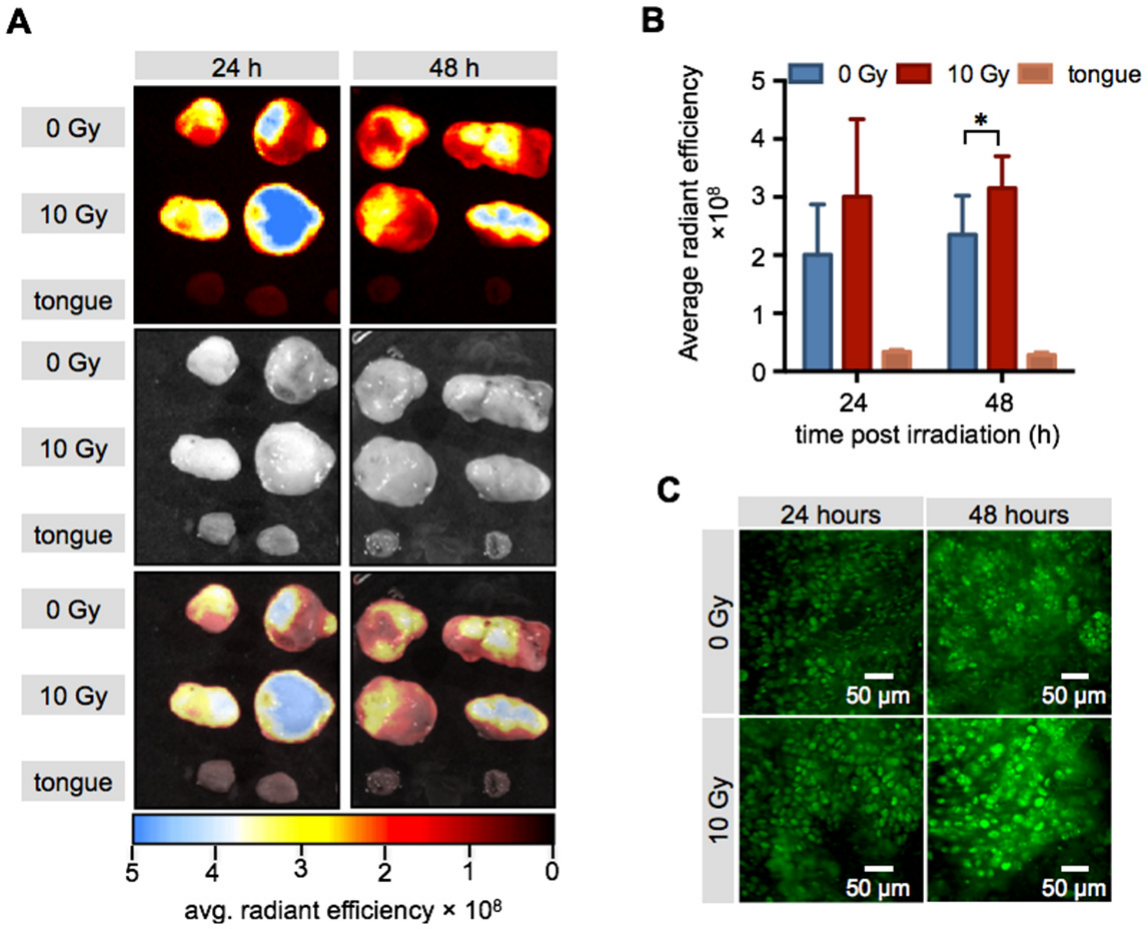
Epifluorescence imaging of PARPi-FL uptake post irradiation. (**A**) Representative epifluorescence images of excised FaDu xenografts and mouse tongues. Displayed are fluorescence only, photograph only and composite images. (**B**) Quantification of PARPi-FL uptake into non-irradiated (0 Gy) and irradiated (10 Gy) xenografts and mouse tongue (n = 5/time point). Shown are means ± SD. Asterisk indicates a p-value of <0.05 using Student’s t-test. (**C**) Representative confocal images of the tumors displayed in (**A**). green: PARPi-FL fluorescence signal.

Determining the uptake of PARPi-FL macroscopically in freshly excised tumor tissues at 24 and 48 hours after irradiation, we found an increased uptake of PARPi-FL, mirroring the pattern of PARP1 protein expression (Fig 7). Specifically, epifluorescence imaging of excised FaDu tumors revealed a statistically significant increase of the average radiant efficiency 48 hours after external beam irradiation (radiance levels were 2.3 ± 0.7×10^8^ and 3.2 ± 0.6×10^8^ for non-irradiated and irradiated tumors 48 hours after treatment, Fig 7A and 7B; p = 0.047), while uptake of PARPi-FL into the normal tongue was negligible (0.3 ± 0.05×10^8^). This trend was also observed on the microscopic level, using confocal microscopy (Fig 7C). Here, too, the strongest nuclear fluorescence was observed in tumors at 48 hours after 10 Gy irradiation. To assess the influence of autofluorescence on the signal in the green fluorescence channel we also looked at a FaDu tumor, which had not received PARPi-FL. Here, we found a low autofluorescence signal with a very narrow histogram, as opposed to the PARPi-FL containing tumor, which displayed a right shift of the histogram curve and much broader distribution of the fluorescent intensities (S2A Fig). To ensure that the PARPi-FL staining is specific to the PARP1 protein, we co-stained cryosections with PARP1 antibody and correlated the PARPi-FL and PARP1 signal. To exclude the possibility of an increase of nonspecific staining after irradiation we chose a tumor that was irradiated with 10 Gy 48 hours prior to PARPi-FL staining, and found a specific localization of the PARPi-FL signal in nuclei (Hoechst staining) which express PARP1 (S2B Fig).

## Discussion

Visualizing and quantifying the amount of external beam radiation delivered to a particular tissue compartment is a major challenge in radiation oncology research [37]. Current methods used for determining the amount of radiation deposited in a given compartment largely rely on theoretical models and externally measured beam intensities [38]. It has been recognized that such models have experienced considerable advances in past decades, but are also becoming prone to error with increasing complexity [39]. Here, we aimed to establish a molecular imaging approach using rapid PARP1 targeted fluorescence imaging to yield a reproducible measure of the effects of external beam radiation to oral cancer tissue.

Our data shows that PARP1 is a robust biomarker, and that the agent accumulates selectively in OSCC cells both *in vitro* and *in vivo*, with and without previous irradiation treatment. We also show that PARP1 indeed responds to ionizing radiation, and that this change can be seen with PARPi-FL for both *in vitro* and *in vivo* experiments.

Our imaging approach is based on the strongly elevated PARP1 expression in cancer tissue compared to its healthy surrounding host tissue, as it has been shown for a large number of tumor types [23, 27–29, 40, 41]. Specifically, our xenograft mouse models showed that PARP1 expression was, with levels of 37.2 ± 3.2% and 28.7 ± 1.7% (for FaDu and Cal 27, respectively), 26-fold and 21-fold higher in tumor tissue than tongue tissue (1.4 ± 0.4%, Fig 1). When exposed to up to 10 Gy of external beam radiation, the clonogenic potential of both FaDu and Cal27 was heavily impacted. It was reduced to 2.0 ± 1.0% and 0.6 ± 0.2% for FaDu and Cal27, respectively. In contrast, at short time points after irradiation (between 6 and 48 hours), cells maintained their viability and PARPi-FL staining did not show qualitative differences from non-irradiated cells. Furthermore, specificity of the PARPi-FL uptake was not affected, as shown by blocking studies.

While no changes in PARPi-FL uptake 6 hours after radiation exposure were seen, we found that median PARPi-FL uptake changed at 24 hours, and particularly at 48 hours after an irradiation event. Changes were seen for as little as 2 Gy, and were more pronounced with increasing dose (Fig 3).

The same effect could be observed *in vivo*, where we compared the PARP1 expression of tumors that were treated with radiation to those that did not receive treatment. Specifically, we used mice with bilateral FaDu tumors, one of which was treated with 10 Gy of radiation (Fig 4). In concordance with our *in vitro* experiments, the radiation treatment had a major effect on cell viability, resulting in a tumor volume in the treated group that was 10 times lower than in the non-irradiated tumors (Fig 5). On histological tumor sections, the treatment resulted in an increase of PARP1 expression similar to that seen using flow cytometry *in vitro* (1.5 ± 0.4 fold increase in fluorescence/nucleus in treated versus untreated FaDu xenografts 48 hours post irradiation). Interestingly, not only the expression, but also the density of PARP1 expressing cells increased in tumors at 48 hours after irradiation. This might be due to elevated PARP1 expression in a subset of tumor cells that were expressing low levels of PARP1 before treatment, but could also be a response of the tumor to the irradiation, and resulting immune cell recruitment [42–44]. Ultimately, the increase in PARP1 expression in individual nuclei, paired with higher nuclear densities after irradiation, could also be detected using *ex vivo* whole tumor imaging after PARPi-FL administration (Fig 7). We found that the median radiant efficiency of tumors that were irradiated was 3.2 ± 0.6 × 10^8^ at 48 hours post treatment. Tumors without irradiation had a radiant efficiency of 2.3 ± 0.7 × 10^8^ at 48 hours post treatment. This increase is in the same range as the increase in PARP1 protein expression, suggesting that PARP1 expression levels can be measured on the whole tumor level using PARPi-FL imaging. The selectivity of PARP1 staining *in vivo* was confirmed by confocal imaging, which showed that the overwhelming amount of imaging agent accumulated in cell nuclei, irrespective of whether those were treated with external beam radiation or not. Confocal imaging also showed that 24 and 48 hours after 10 Gy irradiation, staining with PARPi-FL was more intense in individual nuclei, but also more diffuse and diverse (Fig 7C). The irradiation treatment also affects the tumor microenvironment [42], resulting in compromised tumor tissue architecture, paired with dead cells [44], disrupted perfusion [45, 46] and the subsequent effects on tumor penetration and clearance of PARPi-FL in the tumor.

The increased expression of PARP1 post irradiation also becomes interesting considering combination of radiation therapy with PARP1 inhibitor therapy to mediate synthetic lethality to tumor tissue.

In summary, we have shown that PARP1 expression increases in response to external beam radiation, and that this increase can be observed in cell culture and on the tissue level. Further, the fluorescent imaging agent PARPi-FL is able to accumulate in irradiated cell nuclei of tumor tissues, and its accumulation indicates, that it might be possible to use this or other PARP1 targeted imaging agents to delineate tissues exposed to radiation. Future studies will have to elucidate the effects of changing perfusion, cell density and other architectural changes inside the tumor.

Ultimately, the translation of PARP1 imaging to other modalities, for example whole body PET imaging, using ^18^F labeled or dual labeled (^18^F and Bodipy-FL) PARP Inhibitors will be critical to enable clinical PARP1 imaging. We have shown that such probes accumulate in PARP1 expressing tumors specifically [30, 47]. Future investigations will have to clarify the quantitative relationship between PARP1 expression in whole body PET imaging post irradiation and therapy outcome.

## Supporting Information

S1 Fig Specificity control of PARP1 Immunofluorescence staining of irradiated and non-irradiated tumors. Subsequent cryosections of the same tumors were either stained for PARP1 (**A**) or the primary anti-PARP1 antibody was replaced with a nonspecific rabbit IgG (**B**) to assess the extent of nonspecific binding. The secondary goat anti-rabbit antibody was labeled with an AF594 red fluorescent dye. In combination with the primary rabbit anti-PARP1 antibody, nuclear staining can be observed which is absent in the rabbit IgG control. Furthermore, no non-nuclear red fluorescent signals could be observed, indicating that no measurable non-specific binding was induced by the irradiation.
(JPG)

S2 Fig PARPi-FL *ex vivo* imaging specificity control. (**A**) Comparison of the green fluorescence signal (intensity and histogram distribution) in FaDu tumor tissue with and without PARPi-FL injection to assess the potential impact of autofluorescence. (**B**) PARP1 staining of cryosections of a FaDu tumor 48 hours after 10 Gy irradiation to show colocalization between PARPi-FL and PARP1 including specificity controls for the PARP1 staining (replacement of the specific primary anti-PARP1 antibody with rabbit IgG or no primary).
(JPG)

## References

[1] de Arruda FF, Zhung J, Narayana A, Wolden S, Pfister DG, et al (2005) Intensity-modulated radiation therapy (IMRT) for advanced oropharyngeal carcinoma: The MSKCC experience. Journal of Clinical Oncology 23: 503s–503s.

[2] Lee N, Xia P, Fischbein NJ, Akazawa P, Akazawa C, et al (2003) Intensity-modulated radiation therapy for head-and-neck cancer: The UCSF experience focusing on target volume delineation. Int J Radiat Oncol 57: 49–60. 10.1016/s0360-3016(03)00405-x 12909215

[3] Lok BH, Setton J, Caria N, Romanyshyn J, Wolden SL, et al (2012) Intensity-modulated radiation therapy in oropharyngeal carcinoma: effect of tumor volume on clinical outcomes. International journal of radiation oncology, biology, physics 82: 1851–1857. 10.1016/j.ijrobp.2011.03.029 21640497 PMC4978948

[4] Miah AB, Bhide SA, Guerrero-Urbano MT, Clark C, Bidmead AM, et al (2012) Dose-escalated intensity-modulated radiotherapy is feasible and may improve locoregional control and laryngeal preservation in laryngo-hypopharyngeal cancers. International journal of radiation oncology, biology, physics 82: 539–547. 10.1016/j.ijrobp.2010.09.055 21236602

[5] Taylor A and Powell ME (2004) Intensity-modulated radiotherapy—what is it? Cancer imaging: the official publication of the International Cancer Imaging Society 4: 68–73. 18250011 10.1102/1470-7330.2004.0003 PMC1434586

[6] Juarez-Salinas H, Sims JL and Jacobson MK (1979) Poly(ADP-ribose) levels in carcinogen-treated cells. Nature 282: 740–741. 229416 10.1038/282740a0

[7] Benjamin RC and Gill DM (1980) Adp-Ribosylation in Mammalian-Cell Ghosts—Dependence of Poly(Adp-Ribose) Synthesis on Strand Breakage in DNA. Journal of Biological Chemistry 255: 493–501. 7430132

[8] Durkacz BW, Omidiji O, Gray DA and Shall S (1980) (Adp-Ribose)N Participates in DNA Excision Repair. Nature 283: 593–596. 6243744 10.1038/283593a0

[9] Gagne JP, Isabelle M, Lo KS, Bourassa S, Hendzel MJ, et al (2008) Proteome-wide identification of poly(ADP-ribose) binding proteins and poly(ADP-ribose)-associated protein complexes. Nucleic acids research 36: 6959–6976. 10.1093/nar/gkn771 18981049 PMC2602769

[10] Timinszky G, Till S, Hassa PO, Hothorn M, Kustatscher G, et al (2009) A macrodomain-containing histone rearranges chromatin upon sensing PARP1 activation. Nature structural & molecular biology 16: 923–929. 10.1038/nsmb.1664 19680243

[11] Kraus WL and Hottiger MO (2013) PARP-1 and gene regulation: progress and puzzles. Molecular aspects of medicine 34: 1109–1123. 10.1016/j.mam.2013.01.005 23357755

[12] Ahel D, Horejsi Z, Wiechens N, Polo SE, Garcia-Wilson E, et al (2009) Poly(ADP-ribose)-dependent regulation of DNA repair by the chromatin remodeling enzyme ALC1. Science 325: 1240–1243. 10.1126/science.1177321 19661379 PMC3443743

[13] Gottschalk AJ, Timinszky G, Kong SE, Jin J, Cai Y, et al (2009) Poly(ADP-ribosyl)ation directs recruitment and activation of an ATP-dependent chromatin remodeler. Proceedings of the National Academy of Sciences of the United States of America 106: 13770–13774. 10.1073/pnas.0906920106 19666485 PMC2722505

[14] Masson M, Niedergang C, Schreiber V, Muller S, Menissier-de Murcia J, et al (1998) XRCC1 is specifically associated with poly(ADP-ribose) polymerase and negatively regulates its activity following DNA damage. Molecular and cellular biology 18: 3563–3571. 9584196 10.1128/mcb.18.6.3563 PMC108937

[15] El-Khamisy SF, Masutani M, Suzuki H and Caldecott KW (2003) A requirement for PARP-1 for the assembly or stability of XRCC1 nuclear foci at sites of oxidative DNA damage. Nucleic acids research 31: 5526–5533. 14500814 10.1093/nar/gkg761 PMC206461

[16] Rouleau M, Patel A, Hendzel MJ, Kaufmann SH and Poirier GG (2010) PARP inhibition: PARP1 and beyond. Nature reviews Cancer 10: 293–301. 10.1038/nrc2812 20200537 PMC2910902

[17] Hassa PO and Hottiger MO (2008) The diverse biological roles of mammalian PARPS, a small but powerful family of poly-ADP-ribose polymerases. Frontiers in bioscience: a journal and virtual library 13: 3046–3082. 17981777 10.2741/2909

[18] Doly J and Petek F (1966) Etude De La Structure Dun Compose Polyadp-Ribose Synthetise Par Des Extraits Nucleaires De Foie De Poulet. Cr Acad Sci D Nat 263: 1341-&.

[19] Menear KA, Adcock C, Boulter R, Cockcroft XL, Copsey L, et al (2008) 4-[3-(4-cyclopropanecarbonylpiperazine-1-carbonyl)-4-fluorobenzyl]-2H-phthalazin- 1-one: a novel bioavailable inhibitor of poly(ADP-ribose) polymerase-1. Journal of medicinal chemistry 51: 6581–6591. 10.1021/jm8001263 18800822

[20] Deeks ED (2015) Olaparib: first global approval. Drugs 75: 231–240. 10.1007/s40265-015-0345-6 25616434

[21] Bieche I, de Murcia G and Lidereau R (1996) Poly(ADP-ribose) polymerase gene expression status and genomic instability in human breast cancer. Clinical cancer research: an official journal of the American Association for Cancer Research 2: 1163–1167. 9816283

[22] Nosho K, Yamamoto H, Mikami M, Taniguchi H, Takahashi T, et al (2006) Overexpression of poly(ADP-ribose) polymerase-1 (PARP-1) in the early stage of colorectal carcinogenesis. European journal of cancer 42: 2374–2381. 16809031 10.1016/j.ejca.2006.01.061

[23] Ossovskaya V, Koo IC, Kaldjian EP, Alvares C and Sherman BM (2010) Upregulation of Poly (ADP-Ribose) Polymerase-1 (PARP1) in Triple-Negative Breast Cancer and Other Primary Human Tumor Types. Genes & cancer 1: 812–821. 21779467 10.1177/1947601910383418 PMC3092251

[24] Rojo F, Garcia-Parra J, Zazo S, Tusquets I, Ferrer-Lozano J, et al (2012) Nuclear PARP-1 protein overexpression is associated with poor overall survival in early breast cancer. Annals of oncology: official journal of the European Society for Medical Oncology / ESMO 23: 1156–1164. 10.1093/annonc/mdr361 21908496

[25] Sulzyc-Bielicka V, Domagala P, Hybiak J, Majewicz-Broda A, Safranow K, et al (2012) Colorectal cancers differ in respect of PARP-1 protein expression. Polish journal of pathology: official journal of the Polish Society of Pathologists 63: 87–92. 22864776

[26] Salemi M, Galia A, Fraggetta F, La Corte C, Pepe P, et al (2013) Poly (ADP-ribose) polymerase 1 protein expression in normal and neoplastic prostatic tissue. European journal of histochemistry: EJH 57: e13 10.4081/ejh.2013.e13 23807292 PMC3794339

[27] Dziaman T, Ludwiczak H, Ciesla JM, Banaszkiewicz Z, Winczura A, et al (2014) PARP-1 expression is increased in colon adenoma and carcinoma and correlates with OGG1. PloS one 9: e115558 10.1371/journal.pone.0115558 25526641 PMC4272268

[28] Green AR, Caracappa D, Benhasouna AA, Alshareeda A, Nolan CC, et al (2015) Biological and clinical significance of PARP1 protein expression in breast cancer. Breast cancer research and treatment 149: 353–362. 10.1007/s10549-014-3230-1 25528020 PMC4308637

[29] Chow JP, Man WY, Mao M, Chen H, Cheung F, et al (2013) PARP1 is overexpressed in nasopharyngeal carcinoma and its inhibition enhances radiotherapy. Molecular cancer therapeutics 12: 2517–2528. 10.1158/1535-7163.MCT-13-0010 23979918

[30] Carlucci G, Carney B, Brand C, Kossatz S, Irwin CP, et al (2015) Dual-Modality Optical/PET Imaging of PARP1 in Glioblastoma. Molecular imaging and biology: MIB: the official publication of the Academy of Molecular Imaging. 10.1007/s11307-015-0858-0 PMC4609241 25895168

[31] Thurber GM, Yang KS, Reiner T, Kohler RH, Sorger P, et al (2013) Single-cell and subcellular pharmacokinetic imaging allows insight into drug action in vivo. Nature communications 4: 1504 10.1038/ncomms2506 23422672 PMC3579506

[32] Reiner T, Lacy J, Keliher EJ, Yang KS, Ullal A, et al (2012) Imaging Therapeutic PARP Inhibition In Vivo through Bioorthogonally Developed Companion Imaging Agents. Neoplasia 14: 169–IN163. 22496617 10.1593/neo.12414 PMC3323895

[33] Irwin CP, Portorreal Y, Brand C, Zhang Y, Desai P, et al (2014) PARPi-FL—a fluorescent PARP1 inhibitor for glioblastoma imaging. Neoplasia 16: 432–440. 10.1016/j.neo.2014.05.005 24970386 PMC4198695

[34] Menegakis A, Yaromina A, Eicheler W, Dorfler A, Beuthien-Baumann B, et al (2009) Prediction of clonogenic cell survival curves based on the number of residual DNA double strand breaks measured by gammaH2AX staining. International journal of radiation biology 85: 1032–1041. 10.3109/09553000903242149 19895280

[35] Rieckmann T, Tribius S, Grob TJ, Meyer F, Busch CJ, et al (2013) HNSCC cell lines positive for HPV and p16 possess higher cellular radiosensitivity due to an impaired DSB repair capacity. Radiotherapy and oncology: journal of the European Society for Therapeutic Radiology and Oncology 107: 242–246. 23602369 10.1016/j.radonc.2013.03.013

[36] Munshi A, Hobbs M and Meyn RE (2005) Clonogenic cell survival assay. Methods in molecular medicine 110: 21–28. 15901923 10.1385/1-59259-869-2:021

[37] Ling CC, Humm J, Larson S, Amols H, Fuks Z, et al (2000) Towards multidimensional radiotherapy (MD-CRT): biological imaging and biological conformality. International journal of radiation oncology, biology, physics 47: 551–560. 10837935 10.1016/s0360-3016(00)00467-3

[38] Xu XG, Bednarz B and Paganetti H (2008) A review of dosimetry studies on external-beam radiation treatment with respect to second cancer induction. Physics in medicine and biology 53: R193–241. 10.1088/0031-9155/53/13/R01 18540047 PMC4009374

[39] Klein EE, Drzymala RE, Purdy JA and Michalski J (2005) Errors in radiation oncology: a study in pathways and dosimetric impact. Journal of applied clinical medical physics / American College of Medical Physics 6: 81–94. 16143793 10.1120/jacmp.v6i3.2105 PMC5723492

[40] Galia A, Calogero AE, Condorelli R, Fraggetta F, La Corte A, et al (2012) PARP-1 protein expression in glioblastoma multiforme. European journal of histochemistry: EJH 56: e9 10.4081/ejh.2012.e9 22472897 PMC3352138

[41] Staibano S, Pepe S, Lo Muzio L, Somma P, Mascolo M, et al (2005) Poly(adenosine diphosphate-ribose) polymerase 1 expression in malignant melanomas from photoexposed areas of the head and neck region. Human pathology 36: 724–731. 16084940 10.1016/j.humpath.2005.04.017

[42] Russell JS and Brown JM (2013) The irradiated tumor microenvironment: role of tumor-associated macrophages in vascular recovery. Frontiers in physiology 4: 157 10.3389/fphys.2013.00157 23882218 PMC3713331

[43] De Palma M and Lewis CE (2013) Macrophage regulation of tumor responses to anticancer therapies. Cancer cell 23: 277–286. 10.1016/j.ccr.2013.02.013 23518347

[44] Lauber K, Ernst A, Orth M, Herrmann M and Belka C (2012) Dying cell clearance and its impact on the outcome of tumor radiotherapy. Frontiers in oncology 2: 116 10.3389/fonc.2012.00116 22973558 PMC3438527

[45] Park HJ, Griffin RJ, Hui S, Levitt SH and Song CW (2012) Radiation-induced vascular damage in tumors: implications of vascular damage in ablative hypofractionated radiotherapy (SBRT and SRS). Radiation research 177: 311–327. 22229487 10.1667/rr2773.1

[46] Ren Y, Fleischmann D, Foygel K, Molvin L, Lutz AM, et al (2012) Antiangiogenic and radiation therapy: early effects on in vivo computed tomography perfusion parameters in human colon cancer xenografts in mice. Investigative radiology 47: 25–32. 10.1097/RLI.0b013e31823a82f6 22178893 PMC4446123

[47] Carney B, Carlucci G, Salinas B, Di Gialleonardo V, Kossatz S, et al (2015) Non-invasive PET Imaging of PARP1 Expression in Glioblastoma Models. Molecular imaging and biology: MIB: the official publication of the Academy of Molecular Imaging. 10.1007/s11307-015-0904-y PMC4841747 26493053

