# Supplementary figures and images for: Optical Imaging of PARP1 in Response to Radiation in Oral Squamous Cell Carcinoma
Source: PLoS One. 2016 Jan 25;11(1):e0147752. doi: 10.1371/journal.pone.0147752 (PMC4726809; doi:10.1371/journal.pone.0147752)

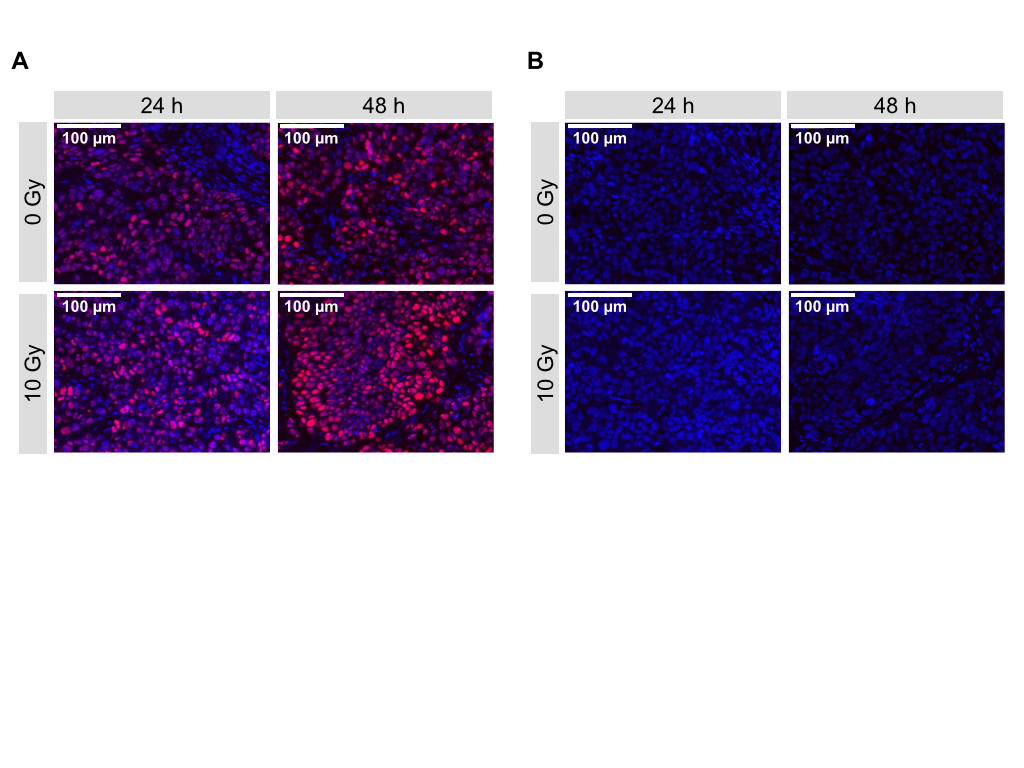

Supplement: S1 Fig — Subsequent cryosections of the same tumors were either stained for PARP1 (A) or the primary anti-PARP1 antibody was replaced with a nonspecific rabbit IgG (B) to assess the extent of nonspecific binding. The secondary goat anti-rabbit antibody was labeled with an AF594 red fluorescent dye. In combination with the primary rabbit anti-PARP1 antibody, nuclear staining can be observed which is absent in the rabbit IgG control. Furthermore, no non-nuclear red fluorescent signals could be observed, indicating that no measurable non-specific binding was induced by the irradiation. (JPG) [file pone.0147752.s001.jpg]

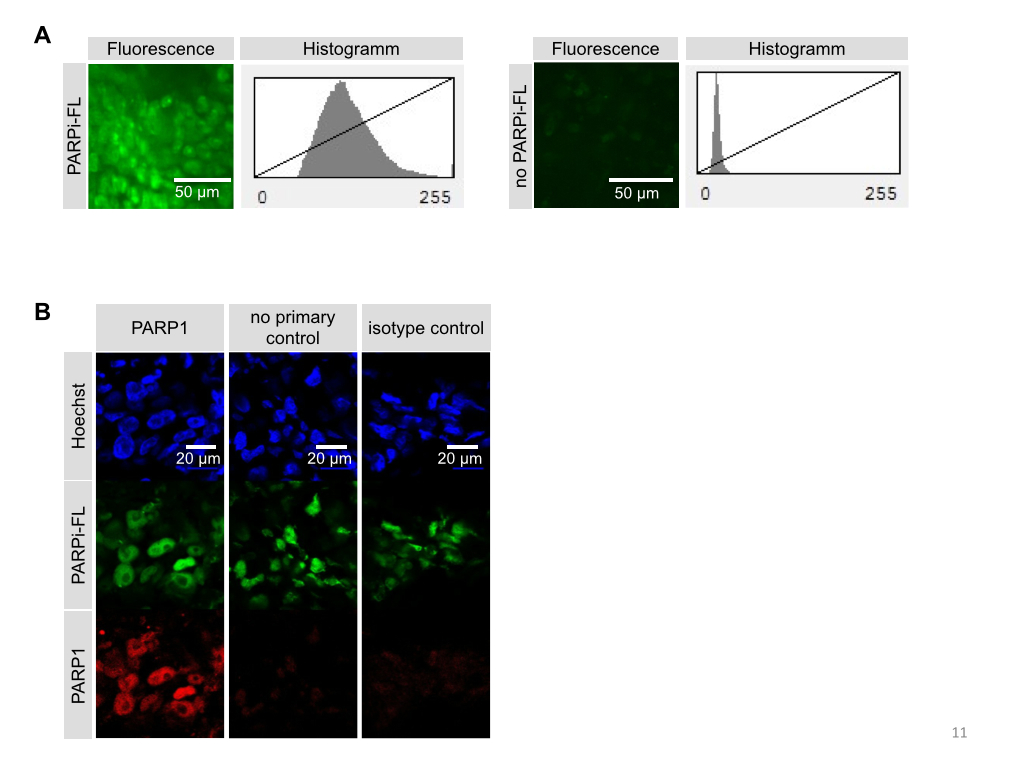

Supplement: S2 Fig — (A) Comparison of the green fluorescence signal (intensity and histogram distribution) in FaDu tumor tissue with and without PARPi-FL injection to assess the potential impact of autofluorescence. (B) PARP1 staining of cryosections of a FaDu tumor 48 hours after 10 Gy irradiation to show colocalization between PARPi-FL and PARP1 including specificity controls for the PARP1 staining (replacement of the specific primary anti-PARP1 antibody with rabbit IgG or no primary). (JPG) [file pone.0147752.s002.jpg]
